# Microbiota-derived propionate suppresses *Salmonella* virulence gene expression via LuxS quorum sensing

**DOI:** 10.1186/s40168-026-02366-0

**Published:** 2026-02-19

**Authors:** Qianyun Zhang, Qidong Zhu, Yunqi Xiao, Shiyong Liao, Shangzhou Liu, Shourong Shi

**Affiliations:** Jiangsu lnstitute of Poultry Science, Yangzhou, 225125 China

**Keywords:** Propionate, Quorum sensing, *Salmonella*, Virulence gene expression, LuxS

## Abstract

**Background:**

Despite mounting evidence that commensal microbes enhance host defenses, whether and how they directly suppress pathogen virulence remains elusive. Here, we investigate metabolites from the gut microbiota of infection‑resistant Tibetan chickens for their ability to reduce *Salmonella* virulence gene expression and elucidate the molecular mechanism by which these compounds inhibit the LuxS/AI‑2 quorum‑sensing system.

**Results:**

Initially, we compared the expression of the quorum‑sensing gene *luxS* and biofilm-associated virulence genes in Tibetan chickens and broiler chickens post-*Salmonella* infection. Notably, Tibetan chickens exhibited significantly lower virulence gene expression than broiler chickens. Subsequently, fecal microbiota transplantation (FMT) from Tibetan chickens to broiler chickens reduced virulence gene expression in infected recipients. Further, 16S rRNA gene sequencing of cecal contents revealed that FMT enhanced microbial diversity and altered composition in infected broiler chickens, specifically enriching short-chain fatty acids (SCFA)-producing beneficial bacteria (e.g., *Bacteroides*, *Rikenellaceae*_*RC9*_*gut*_*group*, *Phascolarctobacterium*, *Desulfovibrio*). Critically, using Transwell chambers to separate microbes and metabolites, we identified metabolites as mediators of this effect. Subsequent liquid chromatography-mass spectrometry (LC–MS) quantification demonstrated significantly elevated propionate concentrations in both uninfected and infected Tibetan chickens, and FMT-recipient broiler chickens. Propionate levels correlated negatively with key virulence factor expression. Moreover, in vitro experiments showed that propionate inhibited *Salmonella* biofilm formation, reduced autoinducer-2 (AI-2) activity, and downregulated the expression of virulence genes. In vivo, we further confirmed that propionate decreased the expression of *Salmonella* virulence genes. Taken together, these results support that propionate suppresses *Salmonella* virulence gene expression by targeting the LuxS/AI-2 quorum-sensing pathway. To validate this mechanism, we generated a *luxS* knockout strain by homologous recombination; strikingly, propionate failed to attenuate virulence gene expression in this mutant, thereby establishing the essential role of LuxS/AI-2. Finally, molecular docking identified propionate-LuxS binding sites (Ile53), and site-directed mutagenesis validated critical functional residues, highlighting structural determinants for virulence gene expression regulation.

**Conclusion:**

These findings underscore the role of the gut-derived metabolite propionate in directly modulating pathogen virulence gene expression by targeting the LuxS/AI-2 quorum‑sensing system, offering novel insights into microbiota-based strategies for infectious disease management.

**Graphical Abstract:**

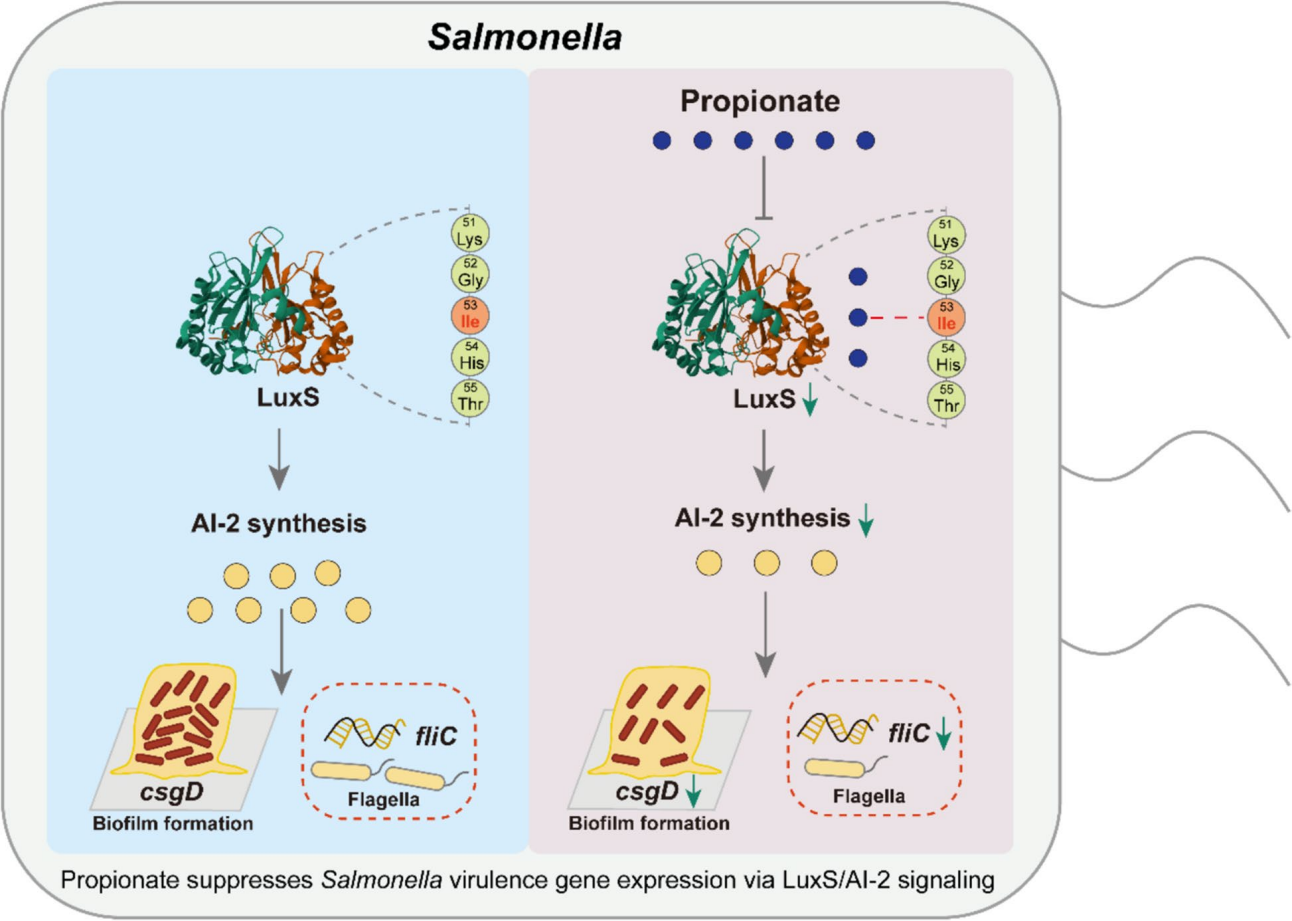

**Supplementary Information:**

The online version contains supplementary material available at 10.1186/s40168-026-02366-0.

## Introduction

Distinct metabolomics profiles are increasingly recognized as hallmarks of host populations with enhanced resistance to infectious diseases [1, 2]. For instance, in chickens infected with *Salmonella enterica* and stratified by the heterophil-to-lymphocyte (H/L) ratio, a heritable immune trait, birds with a low H/L ratio exhibit greater resistance and higher cecal short-chain fatty acid levels than those with a high H/L ratio [3]. Similarly, in a dextran sulfate sodium (DSS)-induced colitis model, the intestines of disease-resistant Min pigs harbor higher concentrations of short-chain fatty acids (SCFAs) and indoles derivatives compared to the more susceptible Yorkshire breed [1]. In murine models, B6N mice exhibit significantly greater resistance to *Salmonella* infection than 129 mice, a phenotype associated with a higher abundance of gut *Bacteroides* and elevated intestinal levels of propionate [4]. These findings collectively suggest that specific host-derived or microbiota-modulated metabolites play a central role in modulating susceptibility to infection and maintaining intestinal homeostasis. As such, elucidating the mechanistic links between host-specific metabolic landscapes and diseases resistance represents a critical frontier in the development of metabolite-based therapeutic and preventive strategies.

*Salmonella* is a globally prevalent pathogen responsible for morbidity and mortality, manifesting in diseases ranging from acute self-limiting gastroenteritis to severe systemic infections. In addition to its broad host range and versatile pathogenicity, *Salmonella* is increasingly exhibiting antimicrobial resistance. This complicates both clinical management and infection control on farms, posing major challenges to global public health and food safety [5]. Among the various host- and microbiota-derived factors implicated in pathogen control, SCFA have emerged as key modulators of microbial ecology and host defense. Propionate, a predominant SCFA produced primarily by *Bacteroides* species through anaerobic fermentation of complex carbohydrates, has been shown to enhance colonization resistance against *Salmonella* [4]. In murine models, elevated levels of *Bacteroides* and consequent increases propionate are associated with enhanced resistance to *Salmonella* infection [4]. Mechanistically, propionate has been demonstrated to inhibit *Salmonella* growth by disrupting intracellular pH homeostasis [4]. However, current studies have primarily focused on its bacteriostatic effects, with limited attention given to its potential role in modulating bacterial virulence. Given that pathogenicity is governed not only by microbial proliferation but also by the regulation of virulence gene expression, the extent to which propionate influences *Salmonella* virulence programs remains unknown. Addressing this knowledge gap is essential for a more comprehensive understanding of how microbiota-derived metabolites contribute to host resistance and pathogen control.

Quorum sensing is a pivotal regulatory mechanism in *Salmonella*, coordinating population-wide behaviors that are essential for pathogenicity, including biofilm formation, motility, and host cell invasion [6–8]. Central to this system is the *luxS* gene [9], which encodes *S*-ribosylhomocysteine lyase-an enzyme required for the synthesis of autoinducer-2 (AI-2), a conserved signaling molecule involved in interspecies communication [10, 11]. AI-2 functions as a key modulator of virulence-associated phenotypes in *Salmonella*, including the formation of biofilm that confer protection against environmental stressors and antimicrobial agents, as well as the regulation of genes critical for epithelial invasion and intracellular survival [12–14]. Modulating *luxS* expression and AI-2 signaling therefore represents a strategic point of intervention for attenuating *Salmonella* virulence and disrupting key steps in its infection cycle.

This study identifies propionate, a microbiota-derived short-chain fatty acid (SCFA), as a critical modulator of *Salmonella* virulence gene expression. Using a comparative model of Tibetan chickens and commercial broilers, we demonstrate that reduced *Salmonella* virulence gene expression in Tibetan chickens is transferable via fecal microbiota transplantation and is associated with elevated intestinal propionate levels. Functional assays reveal that propionate significantly attenuates *Salmonella* pathogenicity in-vivo and in-vitro, not by inhibiting bacterial growth, but by suppressing the LuxS/AI-2 quorum sensing system-a key regulator of virulence gene expression. Furthermore, structural and mutational analyses identify specific LuxS residues essential for propionate binding and its downstream effects. These findings reveal a potential mechanism by which gut microbial metabolites directly regulate bacterial virulence gene expression, offering new avenues for microbiota-targeted therapeutic strategies against enteric infections.

## Results

### Reduced virulence of S. Enteritidis in indigenous Tibetan chickens is linked to distinct gut microbiota composition and altered expression of key virulence-associate genes

**Fig. 1:**
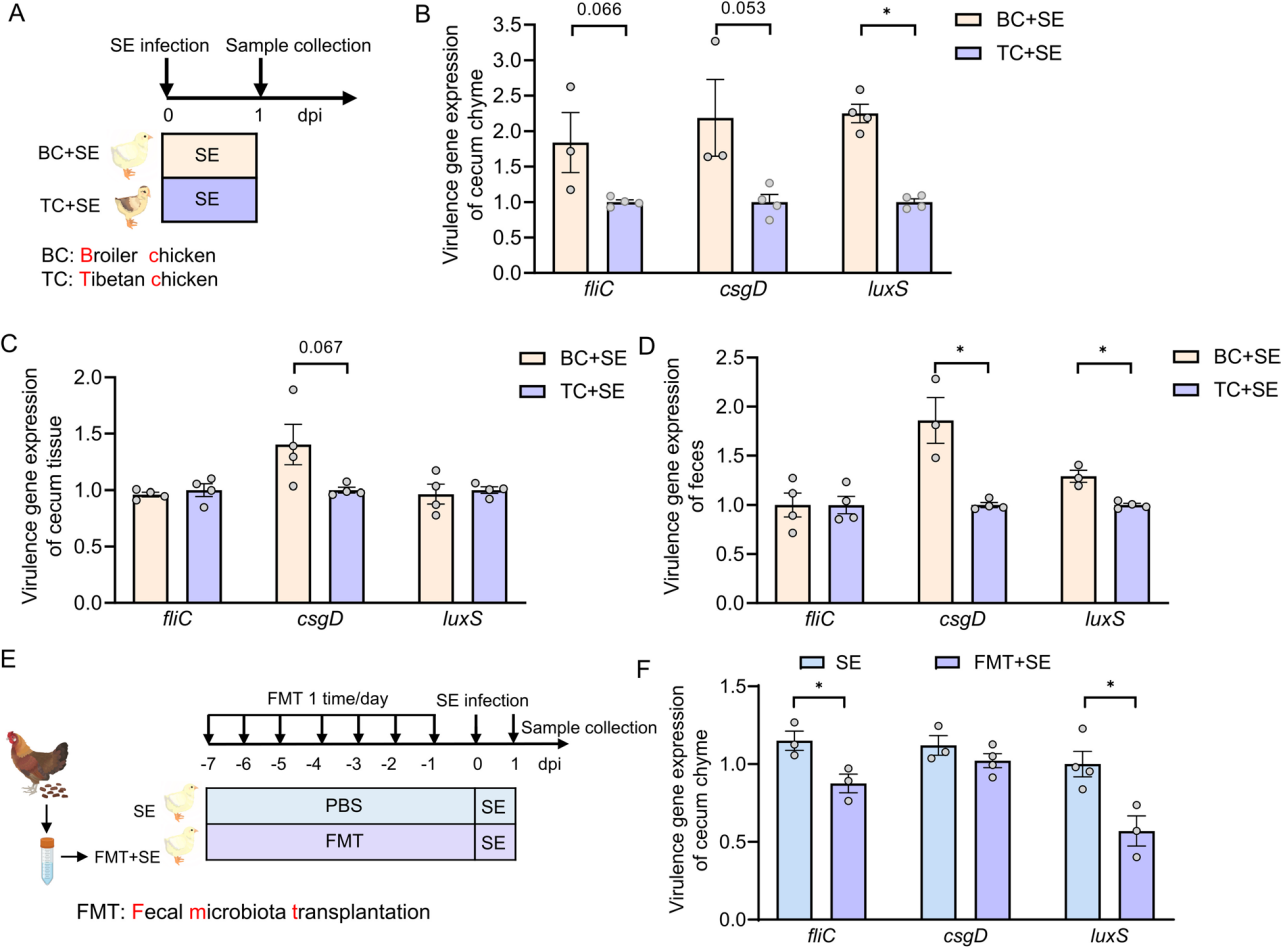
Local Tibetan chickens exhibit reduced *S*. Enteritidis virulence gene expression compared to commercial broilers. **A**–**D** One-day-old broiler chickens (*n* = 8) and Tibetan chickens (*n* = 8) were pre-fed for 7 days and subsequently challenged with *S*. Enteritidis at 1 × 10^11^ CFU/kg body weight (BW). Samples were collected 1-day post-infection. **A** Schematic of the experimental design for panels **B** and **C**. **B** Relative expression levels of *S*. Enteritidis virulence gene in cecal chyme, assessed by qRT-PCR (*n* = 3–4). **C** Relative transcript levels of key *S*. Enteritidis virulence gene in cecal tissue (*n* = 3–4). **D** Relative transcript levels of key *S*. Enteritidis virulence gene in fecal samples (*n* = 3–4). **E**, **F** One-day-old broiler chickens were pre-treated for 6 consecutive days with fecal microbiota from Tibetan chickens and infected on day 7 with *S*. Enteritidis (1 × 10^10^ CFU/mL). Samples were collected at 1-day post infection. **E** Schematic of the experimental design for panels **E** and **F**. **F** Relative expression levels of *S*. Enteritidis virulence genes in cecal chyme after fecal microbiota transplantation (*n* = 3–4). For all panels, data are presented as geometric mean ± SEM. For comparisons between two groups, an unpaired two-tailed Student’s *t* test was used when assumptions of normality and equal variances were met; otherwise, the Wilcoxon rank-sum (Mann–Whitney *U*) test was applied, *P < 0.05. was considered statistically significant

Previous studies have demonstrated that host genetic background influences susceptibility to *Salmonella* infection, with varying colonization capacities observed across different chicken breeds and inbred mouse strain [15–19]. Building on this concept, our prior study showed that co-housing broiler chickens with *Salmonella*-resistant Tibetan chickens increased broiler resistance to *S*. Enteritidis, consistent with the acquisition of protective microbiota from Tibetan chickens [20]. However, the mechanistic basis for breed-dependent variation in *Salmonella* virulence gene expression and colonization remains poorly defined. In particular, the potential role of host-associated gut microbiota in modulating pathogen behavior post-infection warrants further investigation. To address this gap, we compared host responses and pathogen virulence following oral challenge with *S*. Enteritidis in Tibetan chicken and broiler chickens (Fig. 1A). Transcriptional analysis of key *S*. Enteritidis virulence genes—including *luxS* (involved in quorum sensing; *P* < 0.05), *fliC* (encoding flagellin; *P* = 0.066), and *csgD* (a central regulator of biofilm formation; *P* = 0.053)—revealed lower expression in the cecal tissue of infected Tibetan chickens compared to broilers (Fig. 1B). Notably, *csgD* expression was markedly elevated in both the cecum and feces of infected broilers relative to Tibetan chickens (Fig. 1C). Moreover, expression levels of both *csgD* and *luxS* were significantly elevated in the fecal samples of infected broiler chickens (Fig. 1D) suggesting heightened virulence activity in the commercial breed.

These findings indicate differential *S*. Enteritidis virulence gene expression between chicken breeds, with Tibetan chickens being associated with suppressed expression of virulence genes, potentially reflecting host-specific factors that modulate bacterial virulence regulation. Given the established role of gut microbiota in modulating *Salmonella* colonization and virulence [21], we hypothesized that microbiota from Tibetan chickens may contribute to this phenotype. To address this, we performed fecal microbiota transplantation (FMT) by administering fecal material from adult Tibetan chickens to broiler chickens over a 6-day period, followed by *S.* Enteritidis infection (Fig. 1C). Post infection analysis showed that FMT significantly reduced the expression of *fliC* and *luxS* in the cecal contents of recipient broiler compared to PBS-treated controls (Fig. 1D). This result support the notion that Tibetan chickens-derived gut microbiota can attenuate *S*. Enteritidis virulence gene expression in a susceptible host.

### FMT reshapes the cecal microbiota composition in S. Enteritidis‑infected hosts

**Fig. 2:**
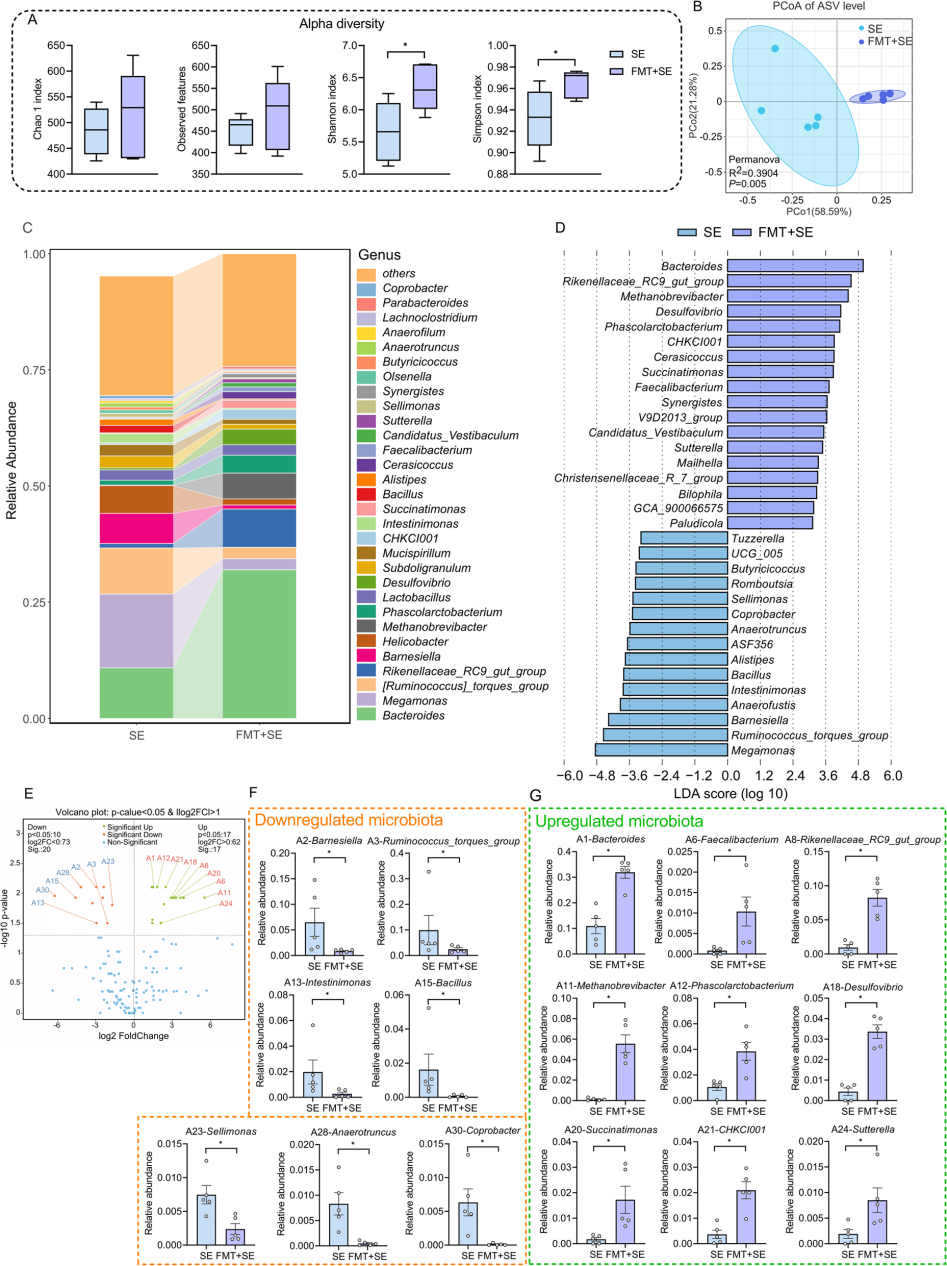
FMT reshapes the cecal microbiota composition in *S*. Enteritidis‑infected hosts. **A** Alpha-diversity analysis, including Chao 1, Observed species, Shannon, and Simpson were performed (*n* = 5). **B** PCoA plot of microbial ASV levels based on Bray analysis, and significance calculated using PERMANOVA (*n* = 5). **C** The top 30 microbial compositions of two groups in the caecum at the genus level (*n* = 5). **D** LEfSe analysis (LDA > 3) was used to identify differential taxa between the two groups (*n* = 5). **E** Volcano plot (*P* < 0.05,|log_2_ fold change| ≥ 1) illustrating differential microbial taxa between the two groups (*n* = 5). **F** Bacterial taxa significantly downregulated in the FMT+SE group compared to the SE group (*n* = 5). **G** Bacterial taxa significantly upregulated in the FMT+SE group compared to the SE group (*n* = 5). For all panels, data are presented as geometric mean ± SEM. For comparisons between two groups, an unpaired two-tailed Student’s *t* test was used when assumptions of normality and equal variances were met; otherwise, the Wilcoxon rank-sum (Mann–Whitney *U*) test was applied, *P < 0.05. was considered statistically significant

To investigate the impact of FMT on the cecal microbiota composition in hosts infected with *S*. Enteritidis, we analyzed the cecal microbiome. Our results indicated that FMT did not significantly alter richness-related alpha diversity, as reflected by comparable observed features and chao1 indices estimates between groups. In contrast, FMT significantly increased abundance-weighted alpha diversity, as evidenced by higher Shannon and Simpson indices, indicating a shift toward a more even community structure with reduced dominance of highly abundant taxa (Fig. 2A). Principal coordinate analysis (PCoA) revealed distinct structural differences between FMT-treated and untreated *S*. Enteritidis-infected groups, confirming FMT-induced reshaping of cecal microbiota composition (Fig. 2B). Genus-level analysis indicated that FMT reduced the relative abundance of potentially harmful bacteria such as *Megamonas* while increasing that of beneficial bacteria such as *Bacteroides* (Fig. 2C). LEfSe analysis (LDA = 3) showed significant enrichment of genera including *Bacteroides*, *Desulfovibrio*, *Phascolarctobacterium*, and *Faecalibacterium* in the FMT group, whereas *Megamonas*, *Barnesiella*, *Anaerofustis*, and *Intestinimonas* were significantly depleted in the SE group (Fig. 2D). Wilcoxon rank-sum test analysis identified 10 significantly downregulated and 17 upregulated genera (Fig. 2F) Consistent with LEfSe results, the differentially abundant genera (top 30) showed downregulation of *Barnesiella*,* Ruminococcus*_*torques*_*group*, *Intestinimonas*, *Bacillus*, *Sellimonas*, *Anaerotruncus*, and *Coprobacter* (Fig. 2E), and upregulation of *Bacteroides*, *Faecalibacterium*, *Rikenellaceae*_*RC9*_*gut*_*group*, *Methanobrevibacter*,* Phascolarctobacterium*, *Desulfovibrio*, *Succinatimonas*, *CHKCI001*, and *Sutterella* (Fig. 2G). Collectively, FMT reshapes the host’s cecal microbiota, thereby reducing expression of the *S*. Enteritis’s virulence factors.

### Tibetan chicken-derived propionate is a key metabolite that suppresses S. Enteritidis virulence gene expression

To identify factors contributing to reduced *S*. Enteritidis virulence gene expression in Tibetan chickens, we investigated the role of gut-derived microbial metabolites. Fecal bacterial suspensions from Tibetan and broiler chickens were co-cultured with *S*. Enteritidis, and biofilm formation was assessed at 4 and 18 h. Co-cultivation with Tibetan chicken-derived fecal bacteria significantly suppressed biofilm formation by *S*. Enteritidis compared to that observed in broiler-derived samples (Fig. S1A), suggesting the presence of microbiota or metabolites capable of modulating bacterial virulence gene expression.

**Fig. 3:**
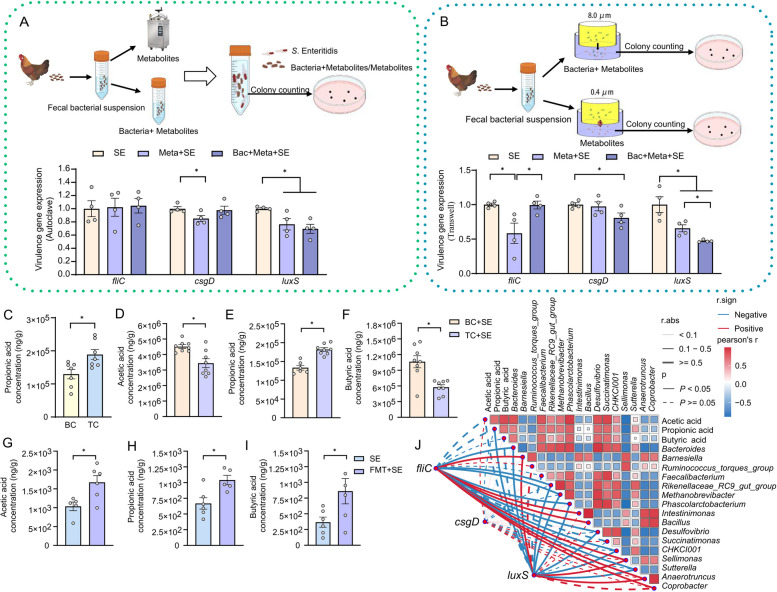
Propionate derived from Tibetan chicken fecal microbiota may mediate suppression of *S*. Enteritidis virulence. **A** Heat-inactivation of Tibetan chicken fecal microbiota was performed to assess whether bacterial viability is essential for reducing *S*. Enteritidis virulence. Expression of virulence genes was quantified by qRT-PCR following co-incubation (*n* = 4). **B** Bacteria and metabolites from Tibetan chickens’ fecal microbiota were separated using a Transwell co-culture system. In the 8 μm pore condition, both bacteria and metabolites could interact with *S*. Enteritidis in the lower chamber; in the 0.4 μm pore condition, only metabolites were allowed to pass through. *S*. Enteritidis virulence gene expression was analyzed after 4 h of co-culture (*n* = 4). **C** Concentration of propionic acid in cecal content of Tibetan chickens and broiler chickens (*n* = 7). **D**–**F** Quantification of short-chain fatty acids (SCFAs) in cecal chyme from *S*. Enteritidis-infected Tibetan and broiler chickens, including **D** acetic acid, **E** propionic acid, and **F** butyric acid (*n* = 6–8). **G**–**I** SCFA concentrations in serum of broiler chickens following fecal microbiota transplantation (FMT) from Tibetan chickens or control treatment, including **G** acetic acid, **H** propionic acid, and **I** butyric acid (*n* = 5–6). **J** Correlation analysis between serum SCFA levels, microbes, and *S*. Enteritidis virulence gene in broiler chickens post-FMT or control. For all panels, data are presented as geometric mean ± SEM. For comparisons between two groups, an unpaired two-tailed Student’s *t* test was used when assumptions of normality and equal variances were met; otherwise, the Wilcoxon rank-sum (Mann–Whitney *U*) test was applied, *P < 0.05. was considered statistically significant

To distinguish the effects of microbial cells from those of their metabolites, we first heat-inactivated the fecal bacterial suspensions to eliminate live microbes. Co-culture of *S*. Enteritidis with heat-treated Tibetan chicken fecal suspensions led to a reduction in the expression of the quorum sensing gene *luxS* and the biofilm regulator *csgD* compared to untreated suspensions led (Fig. 3A). To further separate microbial components from soluble metabolites, we employed a Transwell system that physically isolated microbes from bacterial culture supernatants. Notably, co-culture with microbial metabolites alone significantly reduced *fliC* and *luxS* expression, while the presence of both metabolites and microbes suppressed *luxS* and *csgD* but had no effect on *fliC* (Fig. 3B). These results pointed to soluble microbial metabolites as mediators of reduced *S.* Enteritidis virulence gene expression.

We found that genera that increased after FMT, such as *Bacteroides* [22], *Faecalibacterium* [23], *Phascolarctobacterium* [24], and *Desulfovibrio* [25], produce SCFAs—including acetate, propionate, and butyrate, through the fermentation of complex polysaccharides. We therefore quantified SCFA levels using liquid chromatography-mass spectrometry (LC–MS) in three groups: uninfected Tibetan and broiler chickens, *S*. Enteritidis-infected broiler and Tibetan chickens, and infected broilers receiving FMT from Tibetan chickens. In uninfected birds, Tibetan chickens exhibited significantly higher cecal concentrations of propionate compared to broilers, while acetate and butyrate levels were not significantly different (Figs. 3C and S1B, C). Upon *S*. Enteritidis infection, propionate levels remained elevated in Tibetan chickens, whereas both acetate and butyrate were significantly reduced compared to infected broiler (Fig. 3D–F). Moreover, FMT treatment in infected broiler chickens led to increased serum levels of butyrate, acetate and propionate (Fig. 3G–I). These results suggest that propionate is a microbiota-derived metabolite contributing to the suppression of *S*. Enteritidis virulence gene expression. To further explore the association between* S*. Enteritidis virulence gene expression, microbiota, and metabolites, we performed correlation analyses. These revealed that propionate concentration and specific microbial taxa-including *Bacteroides*, *Rikenellaceae*_*RC9*_*gut*_*group*, *Methanobrevibacter*, *Phascolarctobacterium*, *Desulfovibrio*, *CHKCI001*, and *Sutterella*-were negatively correlated with the expression of three key virulence genes (*fliC*, *csgD*, and *luxS*) (Fig. 3J, Fig. S1D). Concurrently**,** propionate concentration exhibited a positive correlation** w**ith the abundance of these microorganisms (Fig. 3J). Collectively, these findings support the hypothesis that propionate, a microbial metabolite enriched in Tibetan chickens, plays a critical role in attenuating *S*. Enteritidis virulence gene expression.

### Propionate attenuates S. Enteritidis virulence gene expression through inhibition of quorum sensing and biofilm formation

**Fig. 4:**
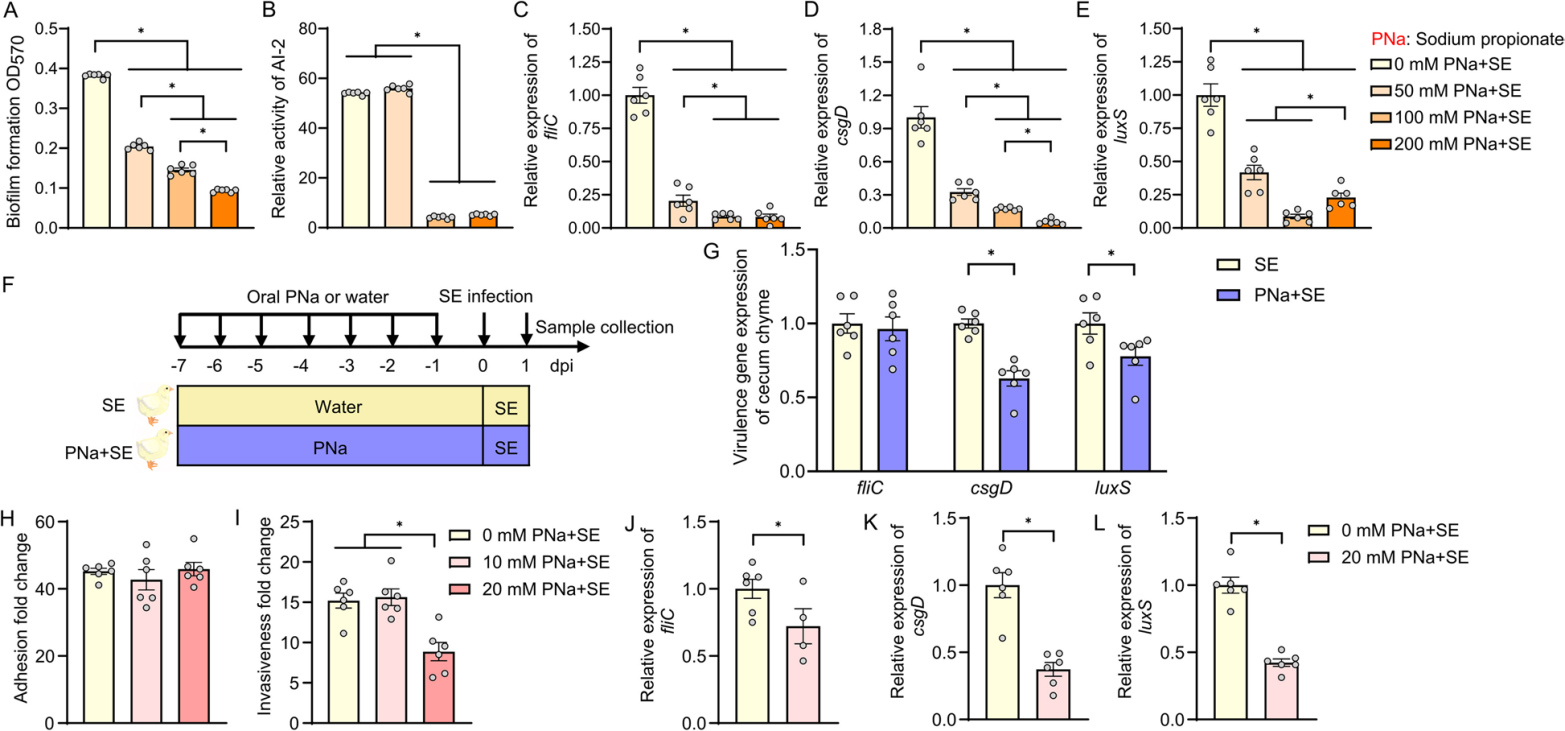
Propionate attenuates *S.* Enteritidis virulence gene expression through suppression of biofilm formation, quorum sensing, and host cell invasion. *S*. Enteritidis was co-cultured with increasing concentrations of sodium propionate to evaluate: **A** biofilm formation and **B** AI-2 quorum sensing activity, quantified via bioluminescence assay (*n* = 6). **C**–**E** Relative transcript levels of virulence-associated genes in *S*. Enteritidis following sodium propionate treatment, including **C** *fliC*, **D** *csgD*, and **E** *luxS* (*n* = 6). **F**, **G** One-day-old broiler chickens were administered sodium propionate in drinking water for 6 days and infected on day 7 with *S*. Enteritidis (1 × 10^10^ CFU/mL). **F** Schematic overview of the experimental design. **G** Relative expression levels of *S. *Enteritidis virulence gene in cecum chyme at 1-day post-infection (*n* = 6). **H**, **I** Assessment of *S. *Enteritidis pathogenesis in Caco-2 cells following sodium propionate pre-treatment: **H** bacterial adhesion and **I** cellular invasion (*n* = 6). **J**–**L** Transcript levels of *S*. Enteritidis virulence genes in infected Caco-2 cells, including **J** *fliC*, **K** *csgD*, and **L** *luxS* (*n* = 6). For all panels, data are presented as geometric mean ± SEM. For comparisons between two groups, an unpaired two-tailed Student’s *t* test was used when assumptions of normality and equal variances were met; otherwise, the Wilcoxon rank-sum (Mann–Whitney *U*) test was applied. For comparisons among three or more groups, one-way analysis of variance (ANOVA) was used when normality and equal-variance assumptions were satisfied; otherwise, the Kruskal–Wallis test was used. *P < 0.05. was considered statistically significant

To directly assess the effect of propionate on *S*. Enteritidis virulence gene expression, we first performed in-vitro co-culture experiments using increasing concentrations propionate. Growth curve analysis revealed that *S*. Enteritidis proliferation was completely inhibited at 400 mM, and significantly suppressed at 200 mM propionate (Fig. S2A). Biofilm formation assays demonstrated a dose-dependent reduction in biofilm biomass across the 50–200 mM concentration range (Fig. 4A).

Given that autoinducer-2 (AI-2), synthesized by LuxS, plays a key role in bacterial virulence, quorum sensing, and biofilm regulation [26, 27], we next examined AI-2 activity in the presence of propionate. *S*. Enteritidis with 100- and 200-mM propionate treatment led to a significant reduction in AI-2 signaling activity (Fig. 4B). Consistently, transcriptional analysis showed that propionate at 50-, 100-, and 200-mM concentrations significantly downregulated the expression of *fliC*, *csgD*, and *luxS* (Fig. 4C–E), indicating suppression of motility, biofilm formation, and quorum sensing pathways.

To evaluate whether propionate exerts similar effects in vivo, we employed a broiler chicken infection model. Based on preliminary safety testing (Fig. S2B), 50 mM propionate was administered via drinking water for six consecutive days prior to *S*. Enteritidis challenge (Fig. 4F). This treatment significantly reduced the expression of *csgD* and *luxS* in cecal chyme post-infection (Fig. 4G), suggesting a suppression of virulence gene expression within the host.

We further explored the impact of propionate using a Caco-2 human epithelial cell infection model. At 20 mM, propionate had no adverse effect on host cell viability (Fig. S2C); while propionate did not alter *S*. Enteritidis adhesion to Caco-2 cells (Fig. 4H), it significantly reduced bacterial invasion (Fig. 4I). Correspondingly, the expression of *fliC*, *csgD*, and *luxS* was markedly suppressed in bacteria isolated from infected cells treated with propionate (Fig. 4J–L). Collectively, these findings demonstrate that propionate suppresses *S.* Enteritidis virulence gene expression both in vitro and in vivo, by downregulating *luxS*-mediated AI-2 signaling and impairing biofilm formation. This supports the hypothesis that propionate acts as a key microbial metabolite in reducing pathogenicity in host environments.

### LuxS/AI-2 pathway mediates propionate-induced attenuation of S. Enteritidis virulence gene expression

To further delineate the molecular mechanism by which propionate suppresses *S*. Enteritidis virulence gene expression, we generated a *luxS* deletion mutant via λ-red recombination (Fig. S3A) and constructed a complementation strain harboring a plasmid-expressed *luxS*. All strains, including wild-type (WT), *luxS* deletion mutant, and the complemented strain, exhibited comparable growth kinetics (Fig. S3B), indicating that *luxS* deletion did not impair overall bacterial viability.

**Fig. 5:**
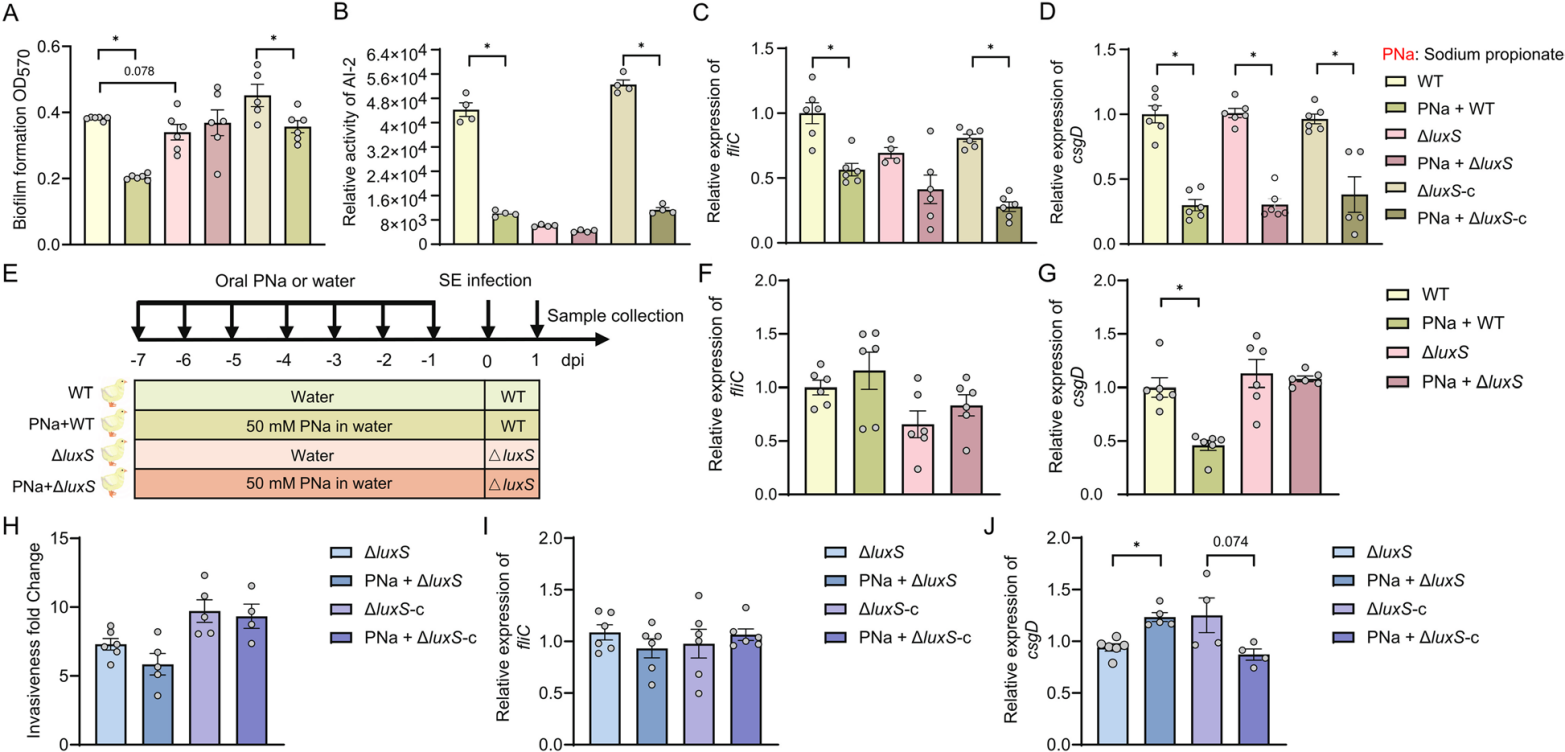
The LuxS/AI-2 quorum sensing system mediates the suppressive effect of propionate on *S.* Enteritidis virulence. **A** The biofilm formation and **B** AI-2 activity of wild-type (WT), *luxS* knockout, and *luxS*-complementation* S*. Enteritidis strains co-cultured with sodium propionate (*n* = 4–6). Relative expression of virulence-associated genes **C** *fliC*, and **D** *csgD* in WT, *luxS *knockout, and complemented *S*. Enteritidis strains following sodium propionate treatment (*n* = 4–6). **E**–**G** One-day-old broiler chickens were treated with sodium propionate for 6 days and infected on day 7 with either WT or *luxS* knockout *S*. Enteritidis (1 × 10^10^ CFU/mL). **E** Schematic representation of experimental design. **F**, **G** Transcript levels of **F** *fliC*, and **G** *csgD* in cecum chime at 1-day post infection (*n* = 6). **H** Invasive capacity of WT and *luxS* knockout *S*. Enteritidis in Caco-2 cells after propionic acid pre-treatment (*n* = 6). **I**, **J** Expression levels of **I** *fliC*, and **J** *csgD* in *S*. Enteritidis-infected Caco-2 cells following propionic acid treatment (*n* = 4–6). For all panels, data are presented as geometric mean ± SEM. For comparisons between two groups, an unpaired two-tailed Student’s *t* test was used when assumptions of normality and equal variances were met; otherwise, the Wilcoxon rank-sum (Mann–Whitney *U*) test was applied, *P < 0.05. was considered statistically significant

Functional assays revealed that *luxS* deletion significantly reduced *S*. Enteritidis biofilm formation and AI-2 activity, both of which were restored upon *luxS* complementation (Fig. S3C, D). Notably, the inhibitory effects of propionate on biofilm formation and AI-2 activity observed in WT strains were abolished in the *luxS* deletion mutant (Fig. 5A, B). Upon complementation, propionate regained its suppressive effects, confirming that the presence of *luxS* is essential for propionate-mediated regulation of virulence gene expression. Gene expression analyses further supported this observation. In the *luxS* deletion mutation background, propionate treatment did not significantly reduce *fliC* expression (*P* = 0.0817), whereas complementation restored the propionate-responsive suppression of *fliC* (Fig. 5C). Interestingly, *csgD* expression remained sensitive to propionate even in the absence of *luxS* (Fig. 5D), suggesting that additional regulatory pathways may mediate propionate’s effect on *csgD* independently of the LuxS/AI-2 system.

In-vivo experiments using the broiler chicken model corroborated the in-vitro findings. Propionate-associated repression of *csgD* during infection was abolished in birds infected with the *luxS* deletion strain (Fig. 5E–G), whereas *fliC* did not show significant suppression under these conditions. Together, these data support LuxS as a key mediator of propionate-responsive anti-virulence gene regulation.

Using a Caco-2 epithelial cell infection model, we found that *luxS* deletion reduced *S*. Enteritidis adhesion and invasion, both of which were restored upon complementation (Fig. S3E, F). When Caco-2 cells were pretreated with 20 mM propionate and subsequently infected with the *luxS* deletion or complemented strains, no significant changes were observed in bacterial invasion or *fliC* expression in the mutant background (Fig. 5H, I). However, *csgD* expression was paradoxically increased after propionate treatment in the *luxS* deletion strain, whereas it was significantly suppressed in the complemented stain (Fig. 5J), again suggesting that *csgD* regulated through both LuxS-dependent and –independent mechanisms.

Taken together, these results demonstrate that the LuxS/AI-2 quorum sensing pathway is a critical mediator of propionate’s inhibitory effect on *S*. Enteritidis virulence gene expression. Specifically, propionate appears to act upstream of *luxS*, suppressing its expression and downstream signaling to reduce motility, invasion, and biofilm formation.

### Propionic acid directly binds to LuxS at isoleucine 53 to suppress S. Enteritidis virulence gene expression

**Fig. 6:**
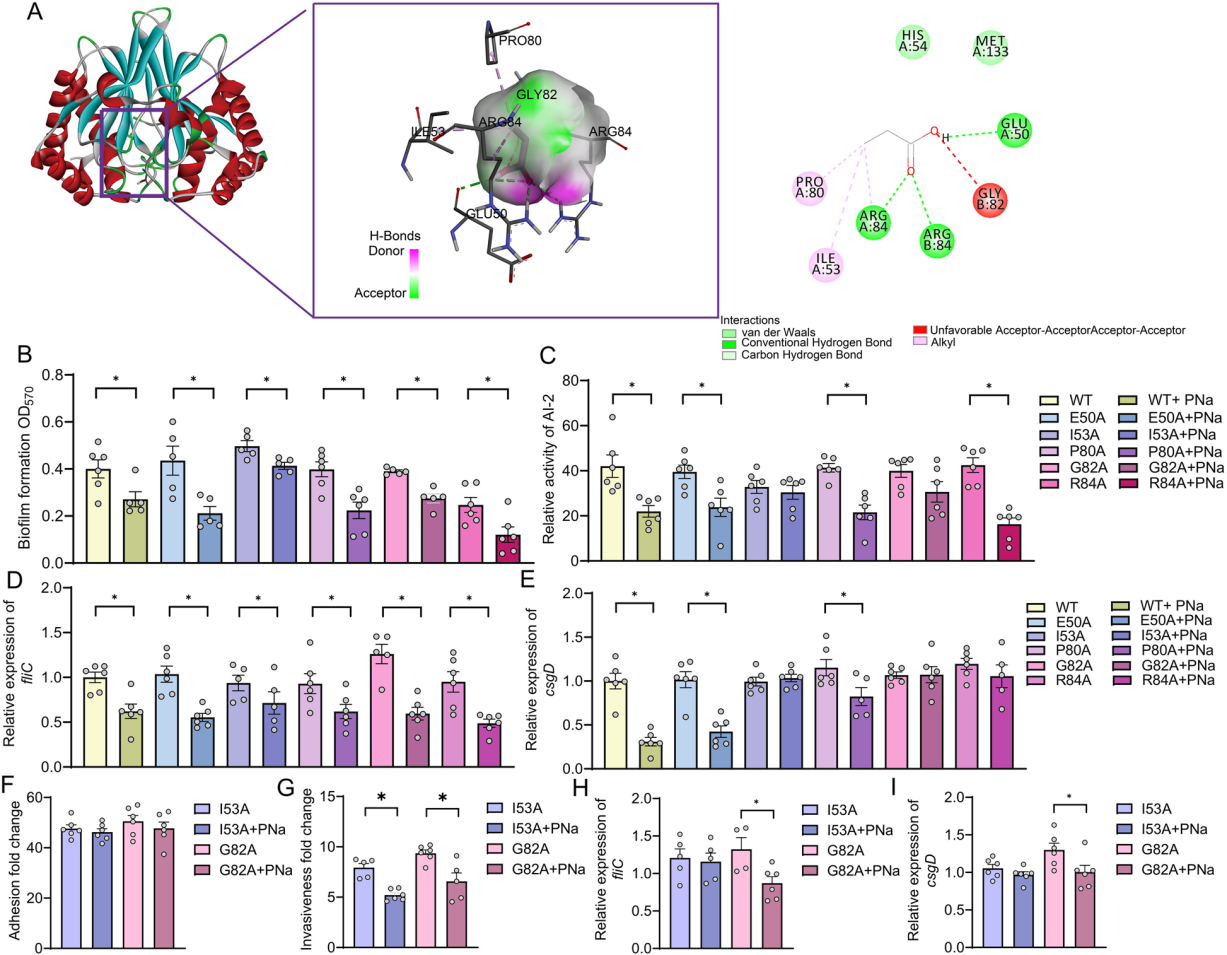
Propionic acid directly interacts with LuxS at a specific binding site to attenuate *S*. Enteritidis virulence. **A** Molecular docking analysis showing direct binding of propionic acid to a specific site on the LuxS protein of *S*. Enteritidis. **B** Biofilm formation capacity and **C** AI-2 quorum sensing activity of *S*. Enteritidis strains expressing LuxS amino acid mutants co-cultured with sodium propionate (*n* = 5–6). **D**, **E** Transcript levels of virulence genes **D** *fliC*, and **E** *csgD* in LuxS mutant strains following sodium propionate acid (*n* = 5–6). **F** Adhesion and **G** invasion capabilities of Caco-2 cells infected with LuxS mutant *S*. Enteritidis stains pre-treated with propionic acid (*n* = 5–6). **H**, **I** Gene expression of **H** *fliC*, and **I** *csgD* in *S*. Enteritidis-infected Caco-2 cells following propionic acid pre-treatment, comparing LuxS mutant strains (*n* = 4–6). For all panels, data are presented as geometric mean ± SEM. For comparisons between two groups, an unpaired two-tailed Student’s *t* test was used when assumptions of normality and equal variances were met; otherwise, the Wilcoxon rank-sum (Mann–Whitney *U*) test was applied, *P < 0.05. was considered statistically significant

To investigate the molecular basis of propionate’s infection with LuxS, we employed molecular docking analysis to predict the binding interface between propionic acid and the luxS protein. The results revealed a strong and specific interaction, characterized by multiple bond types and favorable binding energy. Key residues potentially involved in the binding included Glu50, Ile53, Pro80, Gly82, and Arg84 within the LuxS core domain (Fig. 6A). These residues were further validated by molecular dynamics simulations, which confirmed their spatial proximity and structural conservation, suggesting their importance in ligand interaction and potential regulatory function.

To experimentally validate the docking predictions, we generated site-directed LuxS mutants (E50A, I53A, P80A, G82A, R84A) via alanine substitution at each predicted binding residue. Growth curve analysis showed no significant differences among WT and mutant strains, indicating that the mutations did not impair basic bacterial viability (Fig. S4). Next, we co-cultured each mutant strain with 50 mM propionate in the presence of 1 mM IPTG to induce *luxS* expression and assessed biofilm formation and AI-2 activity. All LuxS mutants showed reduced biofilm formation in response to propionate (Fig. 6B). However, AI-2 signaling activity was not suppressed by propionate in the LuxS^I^^53A^ and LuxS^G82A^ mutants, suggesting disruption of propionate-mediated quorum sensing inhibition at these residues (Fig. 6C). Transcriptional profiling revealed that although *fliC* expression was uniformly suppressed by propionate across all mutants (Fig. 6D), downregulation of *csgD* expression was abrogated in the LuxS^I53A^, LuxS^G82A^, and LuxS^R84A^ strains (Fig. 6E), indicating partial loss of propionate responsiveness.

To further confirm the functional impact of these mutations, we assessed bacterial virulence gene expression using the Caco-2 cell infection model. While adhesion to epithelial cells remained unchanged across all mutants (Fig. 6F), both the LuxS^I53A^ and LuxS^G82A^ mutants displayed reduced invasiveness (Fig. 6G). Notably, only the LuxS^I53A^ strain completely lost responsiveness to propionate, as shown by unchanged *fliC* and *csgD* expression following treatment (Fig. 6H, I), thereby identifying lle53 as a critical residue for propionate-mediated LuxS inhibition.

Together, these findings provide mechanistic evidence that propionate reduces *S*. Enteritidis virulence gene expression by directly binding to LuxS, with isoleucine at position 53 serving as a key interaction site essential for modulating quorum sensing and downstream virulence gene expression.

## Discussion

Indigenous poultry breeds, such as the Tibetan chicken, are increasingly recognized for their robust disease resistance, a trait likely rooted in their distinct genetic composition and gut microbial ecology [1, 28, 29]. In the present study, we leveraged the *S*. Enteritidis infection model to uncover molecular and microbial determinants underlying the differential resistance observed between Tibetan chickens and commercial broilers. Our findings not only corroborate prior reports of enhanced resistance in Tibetan chickens but also delineate a specific microbiota-derived metabolite-propionate that plays a central role in modulating pathogen virulence gene expression via the LuxS/AI-2 quorum sensing system. Collectively, this work provides the first mechanistic evidence that leveraging the microbiota and metabolite profiles of local indigenous breeds can inform strategies to enhance disease resistance in commercial poultry. These findings underscore the translational potential of underexploited genetic and microbial resources in native breeds for improving the health and resilience of industrial livestock populations.

Following challenge with *S*. Enteritidis, Tibetan chickens exhibited reduced expression of *S*. Enteritidis virulence genes. These phenotypes, consistent with their native resilience, were transferable via FMT, suggesting a microbiota-mediated mechanism of resistance [30–34]. Consistent with these interpretations, FMT markedly altered the diversity and composition of the cecal microbiota in infected broilers, indicating that transplantation can rapidly remodel community structure at an early stage of infection, in line with previous reports that FMT mitigates infection-associated dysbiosis [35]. Notably, FMT enriched several genera commonly linked to short-chain fatty acid (SCFA) metabolism, including *Bacteroides* [4], *Faecalibacterium* [36], *Phascolarctobacterium* [37], and *Rikenellaceae*_*RC9*_*gut*_*group* [38], suggesting an increased capacity for SCFA production, particularly propionate. Among the metabolic outputs of the Tibetan chicken microbiota, SCFAs emerged as key modulators, with propionate showing a pronounced capacity to suppress virulence gene expression in vitro and in vivo. Notably, propionate concentrations were consistently elevated in Tibetan chickens regardless of infection status, implicating this metabolite as a predisposing factor in the control of pathogen virulence. These observations align with previous findings that SCFAs, particularly propionate, downregulate *Salmonella* pathogenicity island 1 (SPI-1) expression and impair invasive potential [39].

Mechanistically, our data reveal that propionate inhibits virulence gene expression through repression of the LuxS/AI-2 quorum sensing system-a conserved interspecies communication pathway regulating key pathogenic traits such as biofilm formation, motility, and secretion systems [6, 40, 41]. Specifically, propionate downregulated *luxS* transcription, reduced AI-2 activity, and suppressed the expression of virulence-associated genes including *fliC* and *csgD*. These effects were abolished in *luxS*-deficient strains, firmly establishing LuxS as the primary target of propionate’s action. These results extend earlier studies showing that *luxS* deletion impairs SPI-1 gene expression and attenuates bacterial virulence [42, 43], and provide new evidence that propionate acts upstream of AI-2-mediated signaling in *S*. Enteritidis.

To dissect the molecular underpinnings of this interaction, we employed molecular docking, which predicted stable binding of propionate to the LuxS protein with a binding energy of − 3.9 kcal/mol-more favorable than that of a known LuxS inhibitor ((−)-Dimethyl 2,3-O-isopropylidene-l-tartrate) from *Aeromonas hydrophila* (− 3.06 kcal/mol) [44]. These findings highlight propionate as a promising candidate for targeted inhibition of *S*. Enteritidis virulence. Binding site analysis highlighted lle53, Pro80, and Arg84 as potential interaction residues, with site-directed mutagenesis confirming lle53 as essential for propionate’s inhibitory effect. Mutation of lle53 abolished the ability of propionate to suppress AI-2 production and biofilm gene expression, underscoring the specificity of this interaction. These findings provide compelling mechanistic insight into how a host-derived microbial metabolite can disrupt bacterial communication systems at the molecular level. Importantly, our results are in line with broader literature demonstrating that single-residue substitutions can dramatically alter protein function and microbial phenotypes [45, 46].

Although we identified microorganisms whose abundances positively correlate with propionate concentration in this study, the specific microbial contributors to propionate production in this context remain undefined. The present study focused on the functional role of propionate in attenuating *S*. Enteritidis virulence; however, future work will aim to systematically identify and characterize the propionate-producing microbial taxa within the Tibetan chicken gut ecosystem and explore their ecological interactions. Elucidating these microbial contributors will deepen our understanding of host-microbiota-metabolite interactions and may facilitate the targeted manipulation of gut communities to enhance disease resistance. These efforts hold substantial promise for advancing the strategic utilization of local breed microbiomes in the control of enteric pathogens such as *Salmonella*.

## Conclusions

Together, our study positions propionate as a potent and naturally derived modulator of bacterial virulence gene expression via quorum sensing interference. By identifying a specific microbial metabolite and its molecular target, we bridge the gap between host-microbiota interactions and pathogen control, offering novel avenues for antimicrobial strategies. These insights also emphasize the underutilized potential of indigenous breeds as reservoirs of beneficial microbial functions and metabolites. In a time of rising antimicrobial resistance, such host-microbiota-pathogen interplay presents promising opportunities for the development of microbiome-informed, metabolite-based interventions against enteric infections.

## Experimental section

### Animals

Arbor Acres broiler chickens, a fast-growing commercial line, were obtained from Jiangsu Jinghai Poultry Co., Ltd. (Nantong, China) as 1-day-old chicks (mean body weight, 39.81 ± 0.06 g at arrival). Tibetan chickens were sourced from the Poultry Institute of the Chinese Academy of Agricultural Sciences (Yangzhou, China) as 1-day-old chicks (mean body weight, 26.88 ± 0.20 g at arrival). To minimize confounding variables, birds from both breeds were housed in the same facility and reared under identical standardized husbandry conditions (including stocking density, bedding, temperature, humidity, and lighting schedule), with ad libitum access to feed and water. All birds were fed the same basal diet throughout the study; thus, apart from breed, all experimental conditions were kept consistent. Group sizes for each experiment are provided in the corresponding figure legends. All chickens tested negative for *Salmonella* prior to study initiation. At study termination, all birds were humanely euthanized by cervical dislocation performed by trained personnel, in accordance with the approved protocol of the Animal Care and Use Committee of the Poultry Institute, Chinese Academy of Agricultural Sciences (approval No. CNP20201030).

### Bacterial strains and culture conditions

*S*. Enteritidis strain CMCC50041 was cultured in Luria Bertani broth (LB, Hopebio, China) or on LB agar plates at 37 ℃ with shaking at 180 rpm for 18 h. When necessary, antibiotics were supplemented at the following concentrations: ampicillin (100 μg/mL), kanamycin (50 μg/mL), or chloromycetin (50 μg/mL). For infection assays involving animals or cultured cells, *S*. Enteritidis was grown in modified Martin medium under the same temperature and shaking conditions. Bacterial concentrations for infections were adjusted appropriately prior to use.

For bacterial enumeration, dilutions of cultures or biological samples were plated onto xylose lysine desoxycholate (XLD) (Hopebio, China) agar or deoxycholate hydrogen sulfide lactose (DHL) (Hopebio, China) agar and incubated aerobically at 37 ℃ for 18 h. *Vibrio harveyi* strains BB170 and BB152 were cultured in 2216E liquid medium (Hopebio, China) at 30 ℃ with shaking at 180 rpm for 18 h.

Human epithelial colorectal adenocarcinoma (Caco-2) cells were cultured in complete medium composed of modified Eagle’s medium (MEM) supplemented with 10% fetal bovine serum (FBS; Gibco, USA), 2 mM l-glutamine, and 1% non-essential amino acids). Cells were maintained at 37 ℃ in a humidified incubator with 5% CO_2_. Cells were detached using Trypsin (Pricella, China), seeded into 24-well plates, and incubated for 48 h to reach full confluence prior to experimentation.

### Fecal microbiota transplantation and sodium propionate treatment

For fecal microbiota transplantation (FMT), six adult female Tibetan chickens from the same cohort were selected as fecal donors. Fresh feces samples were collected immediately after defecation, with care taken to remove visible contaminants such as feathers. Samples were collected daily, placed into sterile tubes, and homogenized with sterile PBS (0.01 mol/L) at a ratio of 1:4 (w/v) to prepare a fecal bacteria suspension. The suspension was thoroughly mixed until no visible particulates remained and then centrifuged at 2000 rpm for 1 min at 4 ℃ using a refrigerated centrifuge. A total of 0.1 mL of fecal bacteria suspension was administered to the FMT group for 6 days.

For sodium propionate treatment, sodium propionate (Sigma-Aldrich; ≥ 99% purity) was dissolved in drinking water at the indicated concentration and provided ad libitum for 6 consecutive days.

### S. Enteritidis infection in chickens

For infection experiments, both Tibetan chickens and Arbor Acres broiler chickens (*n* = 8 per treatment group) were orally inoculated at 7 days of age with 0.5 mL of an *S*. Enteritidis suspension. The bacterial concentration was adjusted based on the average body weight of the chickens to achieve an infection dose of 10^11^ CFU/kg body weight.

For broiler chickens undergoing FMT or sodium propionate treatment, infection was performed following 6 consecutive days of pretreatment. At 7 days of age, treated broiler chickens were administered 0.5 mL of *S.* Enteritidis at a concentration of 10^10^ CFU/mL. In specific experiments evaluating the role of LuxS quorum sensing system, a *luxS* knockout strain of *S*. Enteritidis was used in place of the wild-type strain under identical conditions.

### Quantitative real-time analysis of S. Enteritidis virulence genes

Total RNA was extracted from cecal tissue and cecal chime using the RNA-easy isolation reagent kit (Nanjing Vazyme Biotech Co., Ltd., China) according to the manufacturer’s instructions. RNA concentration and purity were assessed using a Nanodrop 2000 micro-spectrophotometer (Thermo Fisher Scientific, USA). For cDNA synthesis, the HiScript two-step RT-qPCR kit (Nanjing Vazyme Biotech Co., Ltd., China) was used, which includes an initial genomic DNA removal step followed by reverse transcription to generate cDNA.

Quantitative real-time PCR was performed using the Hieff ® QPCR SYBR Green Master Mix (Nanjing Vazyme Biotech Co., Ltd., China) on a StepOnePlus™ Real-Time PCR system (Life Technology, USA). The relative expression level of *S*. *Enteritidis* virulence genes in both cecal tissue and chime were quantified using 16*S* *rRNA* as the internal reference. Gene expression was calculated using the 2^−ΔΔCT^ method [47]. Each sample was analyzed in triplicate (technical replicates). Primer sequences used in the analysis are provided in Supplementary Table 1.

### Preparation of fecal bacteria suspensions and co-culture with S. Enteritidis

Following euthanasia, cecal contents were collected aseptically from Tibetan chicken and broiler chickens. Fecal bacterial suspensions were prepared by homogenizing the cecal content with sterile LB broth at a 1:9 (w/v) ratio. The suspension was centrifuged at 2000 rpm for 1 min at 4 ℃, and the resulting supernatant was diluted tenfold with sterile LB to obtain the working fecal bacterial suspension.

To access the interaction between fecal microbiota and *S.* Enteritidis, 10^6^ CFU/mL of *S*. Enteritidis was added to the fecal suspensions derived from either Tibetan or broiler chickens. Cultures were incubated at 37 ℃ with shaking at 180 rpm for 18 h. Biofilm formation was assessed at 4 ℃ and 18 h post-inoculation.

Two methods were used to evaluate the role of metabolites from Tibetan chicken fecal microbiota in modulating *S*. Enteritidis virulence: (1) Heat inactivation: The fecal bacterial suspension from Tibetan chicken was subjected to autoclaving at 121 ℃ for 15 min to inactive microbial cells. Subsequently, 10^6^ CFU/mL *S*. Enteritidis was added to either the inactivated suspension or the non-inactivated (live) fecal suspension, and incubated under standard conditions. (2) Transwell co-culture system: To physically separate bacteria and metabolites, Transwell chambers (Corning, USA) with pore sizes of either 8 μm or 0.4 μm were used. The upper chamber was loaded with Tibetan chicken fecal bacterial suspension, while the lower chamber contained LB broth supplemented with 10^6^ CFU/mL *S*. Enteritidis. The 8-μm membrane permitted the passage of both bacteria and metabolites, whereas the 0.4-µm membrane primarily allows diffusion of small-molecule metabolites while restricting most bacteria. Following 18 h of incubation, *S*. Enteritidis cells from the lower chamber were collected for total RNA extraction. Virulence gene expression was analyzed by quantitative real-time PCR (qRT-PCR) as described previously.

### Sequencing analysis of bacterial colonies and statistical analysis

Total microbial DNA was extracted from the cecal contents of broiler chickens at 1-day post-infection (*n* = 5 per group) using a stool DNA extraction kit (Tiangen Biotech, Beijing, China) according to the manufacturer’s instructions and stored at − 20 °C. DNA concentration and purity were measured using a NanoDrop 2000 spectrophotometer, and DNA integrity was assessed by 2% agarose gel electrophoresis. The V3–V4 region of the bacterial 16S rRNA gene was amplified using primers 338 F and 806R; amplicons were size-verified by agarose gel electrophoresis, purified, and used for library preparation. Libraries were quantified using Qubit, quality-checked by Novogene Co., Ltd. (Beijing, China), and sequenced on an Illumina NovaSeq 6000 platform. Paired-end reads were merged using FLASH (v1.2.11) and quality-filtered with fastp (v0.23.1) to remove low-quality reads and those containing excessive ambiguous bases. Amplicon sequence variants (ASVs) were inferred in QIIME2 (v2025.4) using DADA2 with chimera removal, and taxonomy was assigned against the SILVA v138.1 reference database. Microbial community diversity was assessed using alpha- and beta-diversity metrics. Alpha diversity was quantified using Observed features, Chao1, Shannon, and Simpson indices. Beta diversity was computed using Bray–Curtis dissimilarity, visualized by principal coordinates analysis (PCoA), and tested by PERMANOVA with 9,999 permutations. Genus-level community composition was summarized as relative abundance and visualized using stacked bar plots. Differentially abundant genera between the two groups were identified using linear discriminant analysis effect size (LEfSe) (Wilcoxon rank-sum test with LDA effect size estimation; LDA score > 3 and *P* < 0.05). In addition, differential abundance analysis based on count data was performed using DESeq2, and results were visualized using volcano plots with thresholds of *P* < 0.05 and |log_2_ fold change|≥ 1.

### Short-chain fatty acid quantification by UHPLC-MS/MS

Samples were first flash-frozen in liquid nitrogen and then homogenized with 80% methanol to precipitate proteins. The homogenates were centrifuged at 12,000 rpm for 10 min, and the resulting supernatants were collected. For derivatization, 150 µL of derivatization reagent was added to the supernatant, followed by incubation at 40 ℃ for 40 min. The derivatized samples were then diluted with 80% methanol.

A 95 µl aliquot of each processed sample was mixed with 5 µl of an internal standard solution prior to analysis. Quantification of short-chain fatty acids (SCFAs) was carried out by ultra-high performance liquid chromatography coupled to tandem mass spectrometry (UHPLC-MS/MS) using a Vanquish™ Flex UHPLC system combined with a TSQ Altis™ triple quadrupole mass spectrometer (Thermo Scientific, Germany), conducted by Novogene Co., Ltd. (Beijing, China).

Chromatographic separation was performed using a Waters ACQUITY UPLC BEH C18 column (2.1 × 100 mm, 1.7 μm) maintained at 40 °C. The mobile phase consisted of 10 mM ammonium acetate in water (solvent A) and acetonitrile: isopropanol (1:1, v/v) as solvent B, delivered at a flow rate of 0.30 mL/min.

Standard curves were generated by plotting the concentration ratio of each SCFA standard to the internal standard (X-axis) against the peak area of the SCFA to internal standard (y-axis). Only metabolites with a correlation coefficient (r) > 0.99 was considered for quantification. The limit of quantification (LOQ) was determined based on a signal-to-noise ratio (S/N), using blank matrix comparisons. Final SCFA concentrations in samples were calculated based on the internal standard-corrected standard curves.

### Sodium propionate treatment and S. Enteritidis co-culture

Sodium propionate was prepared at varying concentrations in LB medium and co-culture with *S.* Enteritidis at an initial concentration of 10^6^ CFU/mL. Each treatment group included six biological replicates. Cultures were incubated at 37 ℃ with shaking at 180 rpm for 18 h. After incubation, cultures were centrifuged at 8000 rpm for 5 min. The bacterial pellet was collected for total RNA extraction, while the supernatant was used for autoinducer-2 (AI-2) activity assays.

### Biofilm formation assay

*S*. Enteritidis cultures were growth to an optical density at 600 nm (OD_600_) of 1.0, then diluted 1:100 in fresh LB medium. A total of 200 μL of the diluted bacterial suspension was inoculated into each well of a sterile 96-well microplate. Each strain was tested in six technical replicates. Plates were incubated statically at 37 °C for 24 h to allow biofilm formation.

Following incubation, the culture medium was carefully aspirated, and wells were washed three times with sterile PBS to remove non-adherent bacteria. Plates were then air-dried, and adherent cells were fixed with methanol for 15 min. Methanol was discarded, and the wells were again air-dried. Subsequently, 200 μL of 1% crystal violet solution was added to each well and incubated for 5 min at room temperature. Excess stain was gently rinsed off with distilled water, and the plates were air-dried. To solubilize the retained crystal violet, 200 μL of 33% glacial acetic acid was added to each well and gently shaken. Biofilm formation was quantified by measuring the absorbance at 570 nm (OD_570_) using a microplate reader.

### Detection of AI-2 activity using Vibrio harveyi bioassay

*Vibrio harveyi* BB170 was cultured in sterile 2216E liquid medium until reaching an OD_600_ of 1.0. The culture was then diluted 1:5000 in fresh 2216E medium. For the AI-2 bioassay, 180 μL of the diluted *V*. *harveyi* BB170 culture was mixed with 20 µL of the test sample-previously filtered through a 0.22 μm membrane filter-into each well of a white 96-well microplate. Plates were incubated at 26 ℃ for 4 h under dark conditions. Following incubation, bioluminescence was measured using a microplate reader equipped with luminescence detection capability. AI-2 activity was expressed as a percentage relative to the positive control (*V. harveyi* BB152), using the formula:

AI-2 activity (%) = (Bioluminescence of sample/Bioluminescence of BB152 control) × 100.

### Cell culture

Complete medium for Caco-2 cells was prepared by supplementing MEM with fetal bovine serum (FBS), penicillin–streptomycin, sodium pyruvate, GlutaMax, and non-essential amino acids (NEAA) (80:20:1:1:1:1, v/v). Frozen Caco-2 cells were rapidly thawed in a 37 °C water bath, transferred to a 10-mL tube containing MEM, and centrifuged at 1200 rpm for 3 min. The supernatant was discarded, and the cell pellet was resuspended in complete medium. Cells were seeded into T25 flasks and incubated at 37 ℃ in a humidified atmosphere with 5% CO₂ until ~ 80% confluence. For passaging, cells were washed twice with PBS, detached with 0.25% trypsin-ethylenediaminetetraacetic acid (EDTA), and neutralized with complete medium. Cells were gently resuspended, centrifuged at 1200 rpm for 5 min, resuspended in fresh complete medium, and reseeded into new flasks or used for subsequent experiments.

### Cell viability assay

Cells in the logarithmic growth phase were seeded into 96-well plates at 100 μL per well and incubated at a 37 ℃ with 5% CO_2_ for 24 h to allow adherence. Following incubation, the culture medium was removed and replaced with 100 μL of sodium propionate diluted in MEM to the indicated final concentrations. Each treatment condition was tested in six replicate wells. After 12 h of treatment, 10 μL of cell Counting Kit-8 (CCK-8) reagent was added to each well. Plates were incubated for an additional 2 h at 37 ℃. Cell viability was assessed by measuring absorbance at 450 nm using a microplate reader.

### Caco-2 cell infection assay

Caco-2 cells (10^5^ cells per well) were seeded in 24-well plates and cultured until reaching the logarithmic growth phase. Cells were then treated with various concentrations of sodium propionate (dissolved in MEM) for 4 h, with six replicates per treatment. Following treatment, the medium was removed, and the cells were infected with activated *S*. Enteritidis at a multiplicity of infection (MOI) of 10. After 4 h of incubation at 37 ℃, the infected medium was discarded, and cells were washed twice with PBS to remove non-adherent bacteria.

For the adhesion assay, cells were detached using 0.25% trypsin–EDTA and plated for *S*. Enteritidis enumeration. For the invasion assay, cells were treated with gentamicin solution for 30 min, followed by three washes with PBS to remove all the extracellular antibiotics and bacteria. Cells were then lysed with Triton X-100, and intracellular bacteria were enumerated by plating. In parallel, total RNA was extracted from the infected Caco-2 cells for the analysis of *S.* Enteritidis virulence gene expression using qRT-PCR, as described previously.

### Plasmid construction

Plasmids *p*KD3, *p*KD46, and *p*CP20 were used for the construction of gene knockout of *S*. Enteritidis. Plasmid *p*ET28a was employed for complementation assays in knockout strain. All plasmids were propagated in *Escherichia coli* under selection conditions using appropriate antibiotics corresponding to their resistance markers. Plasmid DNA was extracted using standard plasmid purification protocols and confirmed by Sanger sequencing prior to use in experiments.

### Construction of luxS knockout and complemented strains of S. Enteritidis

The *luxS* gene knockout in *S.* Enteritidis was performed using the λ-red recombination system. Briefly, the chloramphenicol resistance cassette flanked by FRT sites was amplified via PCR using the *p*KD3 plasmid as a template and gene-specific primers containing homologous arms targeting the *luxS* locus. The PCR product was confirmed by agarose gel electrophoresis, and the target fragment was gel-extracted and purified.

The purified fragment was then electroporated into *S.* Enteritidis harboring the pKD46 plasmid, which expresses the λ-red recombinase under arabinose induction. Following electroporation, transformants were selected on LB agar plates supplemented with 50 μg/mL chloramphenicol, confirming insertion of the resistance cassette into the *luxS* locus.

To remove the antibiotic resistance marker, the pCP20 plasmid was introduced into the recombinant strain. The plasmid encodes FLP recombinase, which facilitates excision of the FRT-flanked chloramphenicol resistance cassette. Recombinants were selected on LB agar containing both ampicillin and chloramphenicol, and successful marker excision was confirmed by loss of antibiotic resistance and PCR using gene-specific primers (Supplementary Table 2).

For complementation, the *luxS* coding sequence (wild-type or site-specific mutant) was cloned into the *p*ET-28a(+) vector. The resulting constructs were transformed into electrocompetent *luxS* knockout strains. Transformed bacteria were cultured at 37 ℃ with shaking at 180 rpm for 1 h, then plated on LB agar containing 50 μg/mL kanamycin and incubated overnight at 37 ℃. Colonies were screened for positive clones by PCR and sequence verification.

### Binding sites prediction of LuxS and propionic acid by molecular docking assay

The interaction between propionic acid and LuxS protein was predicted using Schrödinger Suite (Schrödinger LLC, USA). The docking procedure included identification of potential binding sites, evaluation of ligand–protein interactions, and scoring based on predicted binding affinity. LigPrep was used to preprocess the propionic acid molecular, retaining its stereochemical configuration and minimizing its energy using the default MMFFs force field parameters.

The docking simulations were conducted using the Glide XP (Extra Precision) mode to evaluate the binding affinity between propionic acid and the LuxS protein, with docking scores reflecting the strength and stability of ligand-receptor interactions. The binding poses were further analyzed using Maestro for conformational evaluation and binding posture optimization.

Three-dimensional visualization of docking results was performed using PyMOL to access specific molecular interactions, including hydrogen bonds, hydrophobic contacts, and π-π stacking with key residues of the LuxS protein. Additionally, two-dimensional interaction maps were generated using LigPlot+ to illustrate the specific residue-ligand contacts and provide a clear representation of the binding environment.

### Statistical analysis

All data are presented as mean ± standard error of the mean (SEM). Prior to hypothesis testing, data were assessed for normality and homogeneity of variances at the 95% confidence level. Statistical analyses were performed using SPSS Statistics 27.0.1 (IBM Corp., Armonk, NY, USA). For comparisons between two groups, an unpaired two-tailed Student’s *t* test was used when assumptions of normality and equal variances were met; otherwise, the Wilcoxon rank-sum (Mann–Whitney *U*) test was applied. For comparisons among three or more groups, one-way analysis of variance (ANOVA) was used when normality and equal-variance assumptions were satisfied; otherwise, the Kruskal–Wallis test was used. All tests were two-sided, and* P* < 0.05 was considered statistically significant. Unless otherwise specified, graphs were generated using GraphPad Prism 9.5 (GraphPad Software, San Diego, CA, USA).

## Supplementary Information

Supplementary Material 1: Document S1. Figures S1–S4 and Tables S1–S3.

## Data Availability

All 16S rRNA sequencing data generated and analyzed in this study are publicly available in the NCBI Sequence Read Archive (SRA) under the accession number PRJNA1313086. The metabolomics datasets reported in this paper have been deposited in the OMIX repository of the China National Center for Bioinformation/Beijing Institute of Genomics, Chinese Academy of Sciences (https://ngdc.cncb.ac.cn/omix) under the accession numbers OMIX011853 and OMIX011854.
