# Supplementary material for: Microbiota-derived propionate suppresses Salmonella virulence gene expression via LuxS quorum sensing
Source: Microbiome. 2026 Feb 19;14:70. doi: 10.1186/s40168-026-02366-0 (PMC12918161; doi:10.1186/s40168-026-02366-0)
Supplement: Supplementary file 1 — Supplementary Material 1: Document S1. Figures S1–S4 and Tables S1–S3. [file 40168_2026_2366_MOESM1_ESM.docx]

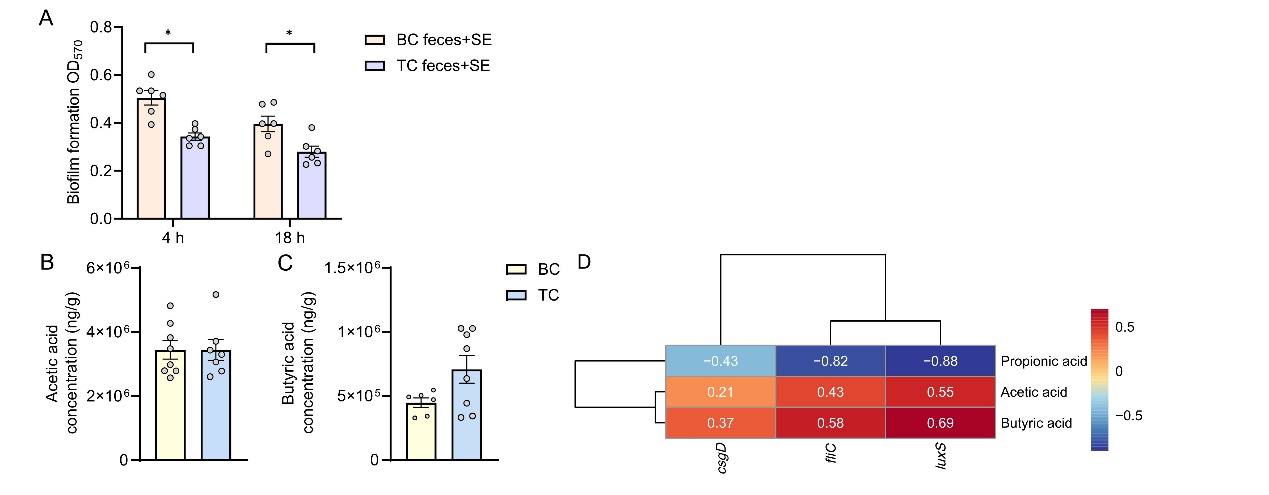


**Figure S1. Propionic acid from Tibetan chicken fecal metabolites underlies the reduced virulence of *S*. Enteritidis, related to Figure 2.**

(A) Biofilm formation of *S*. Enteritidis following of co-culture with fecal microbiota from Tibetan chicken or broiler chickens. (n=6).

Concentration of (B) acetic acid and (C) butyric acid in cecal chyme of Tibetan chickens and broiler chickens.

(D) Correlation analysis of SCFA levels and *S*. Enteritidis virulence gene expression in the cecal chyme of Tibetan chickens and broiler chickens.

For all panels, data are presented as geometric mean ± SEM. For comparisons between two groups, an unpaired two-tailed Student’s t-test was used when assumptions of normality and equal variances were met; otherwise, the Wilcoxon rank-sum (Mann–Whitney U) test was applied, **P* < 0.05. was considered statistically significant.


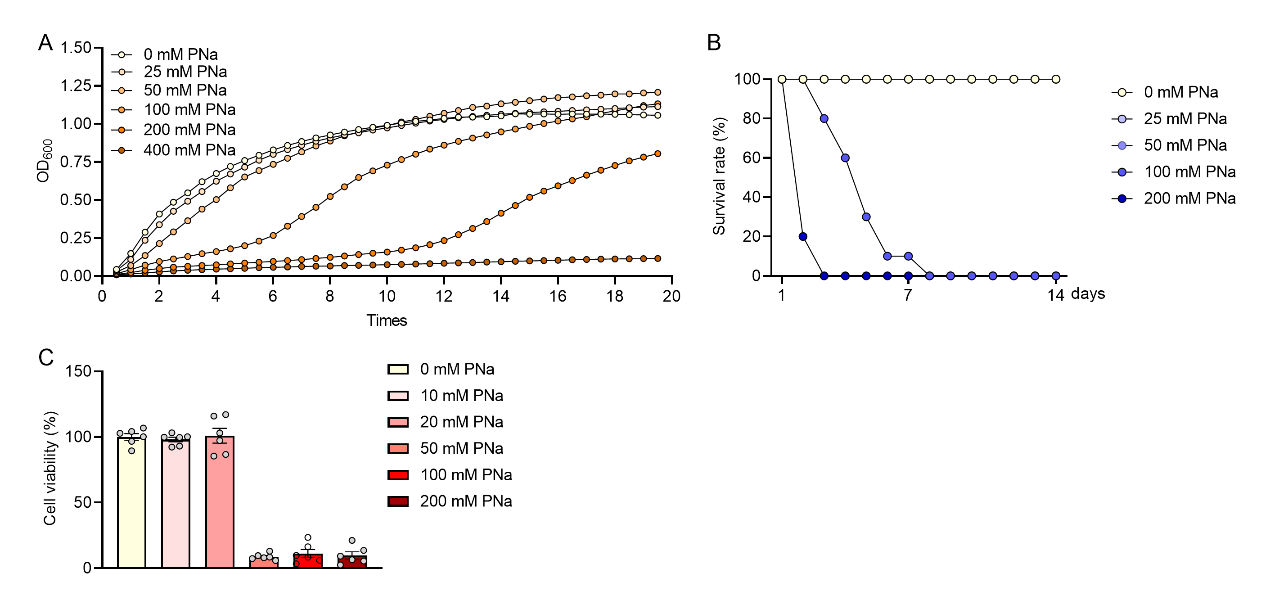


**Figure S2. Determination of non-toxic concentrations of propionic acid for effective action against *S.* Enteritidis, related to Figure 3.**

(A) Growth kinetics of *S.* Enteritidis co-cultured with propionic acid.

(B) Tolerability of propionic acid in broiler chickens.

(C) Cytotoxicity assessment of propionic acid on Caco-2 cells. (n=6).


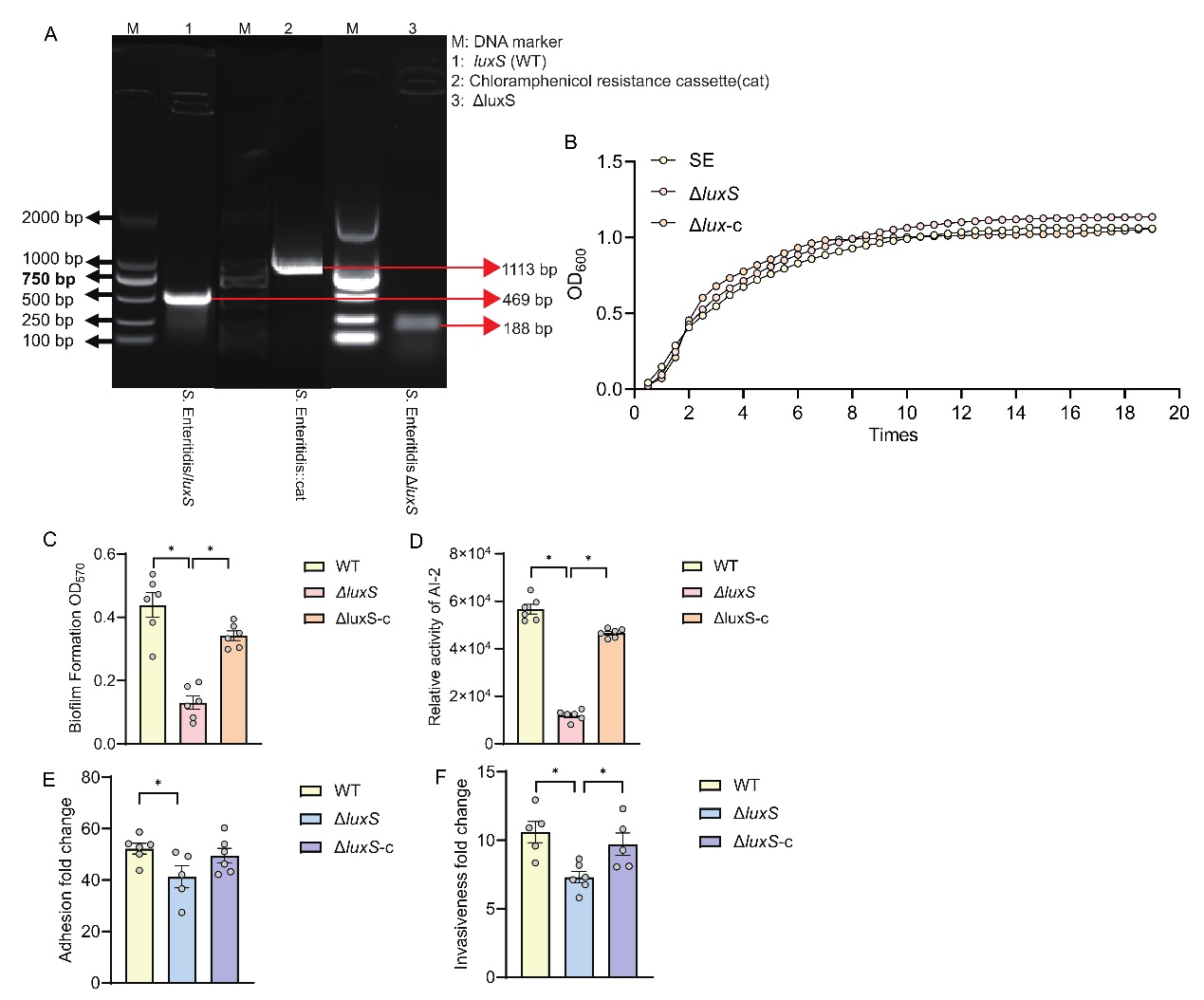


**Figure S3. Knockout of the *luxS* gene attenuates the virulence of *S.* Enteritidis, related to Figure 4.**

(A) Schematic representation of *luxS* gene knockout in *S*. Enteritidis using λ-red homologous recombination.

(B) Growth curves of *S.* Enteritidis wild-type (WT), *luxS* knockout, and *luxS* complementation strains.

(C) Biofilm formation ability and (D) AI-2 quorum sensing activity of wild-type (WT), *luxS* knockout, and complementation strains of *S*. Enteritidis. (n=6).

(E-F) (E) Adhesion and (F) invasion of Caco-2 cells by WT, *luxS* knockout, or complementation strains of *S*. Enteritidis. (n=5-6).

For all panels, data are presented as geometric mean ± SEM. For comparisons between two groups, an unpaired two-tailed Student’s t-test was used when assumptions of normality and equal variances were met; otherwise, the Wilcoxon rank-sum (Mann–Whitney U) test was applied, **P* < 0.05. was considered statistically significant.


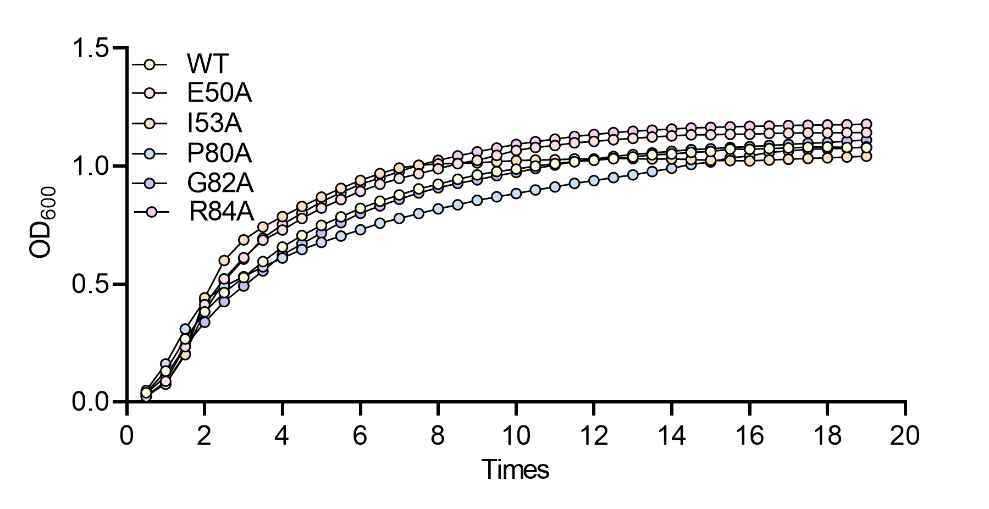


**Figure S4. Amino acid mutations in LuxS do not affect the growth of *S.* Enteritidis, related to Figure 5.**

Growth curve of WT and *LuxS* mutant strains of *S.* Enteritidis, showing comparable proliferation dynamics under standard culture conditions.

**Supplementary table 1** Gene-specific primers for virulence gene amplification

| Genes | Sequences (5′→3′) | Product size（bp） |
| --- | --- | --- |
| *luxS* | TTCGCAGTCGATCATACCCG | 249 |
|  | CATGTAGAAACCGGTACGGC |  |
| *fliC* | ACCAGCTATCGCTTTGGCTT | 255 |
|  | GCGACAGTGGGTGATCTGAA |  |
| *csgD* | ATATCTCAGCCGGTTGCATT | 207 |
|  | CCACGTGTTCCTGGTCTTCA |  |
| *16s* | TTCCAGTGTGGCTGGTCATC | 189 |
|  | TGCCTGATGGAGGGGGATAA |  |

**Supplementary table 2** Primer sequence information for gene editing

| Primer name | Forward primer (5′→3′) | Reverse primer (5′→3′) |
| --- | --- | --- |
| *luxS* | ATAGCTTCGCAGTCGATCAT | TGCAATTCCTGCAGTTTTTC |
| RP | TGCAATTCCTGCAGTTTTTCTTTCGGCAGCGCCAGCTCTTTATTGCTGTTTGTGTAGGCTGGAGCTGCTTCG | ATAGCTTCGCAGTCGATCATACCCGGATGCAAGCGCCGGCGGTCCGGGTTCATATGAATATCCTCCTTAG |
